# Stressful Life Events and Chronic Fatigue Among Chinese Government Employees: A Population-Based Cohort Study

**DOI:** 10.3389/fpubh.2022.890604

**Published:** 2022-07-07

**Authors:** Dan Qiu, Jun He, Yilu Li, Ruiqi Li, Feiyun Ouyang, Ling Li, Dan Luo, Shuiyuan Xiao

**Affiliations:** ^1^Department of Social Medicine and Health Management, Xiangya School of Public Health, Central South University, Changsha, China; ^2^Lixia Center for Disease Control and Prevention of Jinan, Jinan, China; ^3^Mental Health Institute, Second Xiangya Hospital, Central South University, Changsha, China

**Keywords:** stressful life event, self-reported fatigue, working adult, cohort study, Chinese

## Abstract

**Background:**

Currently, evidence on the role of stressful life events in fatigue among the Chinese working adults is lacking. This study aimed at exploring the prospective associations between stressful life events and chronic fatigue among Chinese government employees.

**Methods:**

From January 2018 to December 2019, a total of 16206 government employees were included at baseline and they were followed-up until May 2021. A digital self-reported questionnaire platform was established to collect information on participants' health and covariates. Life events were assessed by the Life Events Scale (LES), fatigue was assessed by using a single item, measuring the frequency of its occurrence. Binary logistic regression analysis was used for the data analysis.

**Results:**

Of the included 16206 Chinese government employees at baseline, 60.45% reported that they experienced negative stressful life events and 43.87% reported that they experienced positive stressful life events over the past year. Fatigue was reported by 7.74% of the sample at baseline and 8.19% at follow-up. Cumulative number of life events at baseline, and cumulative life events severity score at baseline were positively associated with self-reported fatigue at follow up, respectively. After adjusting sociodemographic factors, occupational factors and health behavior related factors, negative life events at baseline (OR: 2.06, 95% CI: 1.69–2.51) were significantly associated with self-reported fatigue at follow-up. Some specific life events including events related to work and events related to economic problems were significantly associated with self-reported fatigue. Specifically, work stress (OR = 1.76, 95%CI: 1.45–2.13), as well as not satisfied with the current job (OR = 1.95, 95%CI: 1.58–2.40), in debt (OR = 1.75, 95%CI: 1.40–2.17) were significantly associated with self-reported fatigue. The economic situation has improved significantly (OR = 0.62, 95%CI: 0.46–0.85) at baseline was significantly associated with lower incidence of self-reported fatigue.

**Conclusion:**

Negative stressful life events were associated with fatigue among Chinese government employees. Effective interventions should be provided to employees who have experienced negative stressful life events.

## Background

As a non-specific symptom often overlooked in primary health care, fatigue refers to self-reported physical and mental tiredness, weakness, and a lack of energy (1). Fatigue is a significant public health concern given its negative health consequences and high prevalence. Prevalence of fatigue in the general population ranged from 0.37% to 18.3% (2–4). People who perceived fatigue often report poor self-care activities, significant social and functional impairment, and great use of general medical services (1, 5, 6). For the working population, fatigue not only affects work performance and productivity, severe fatigue may lead to sick leave and work disability (7, 8).

Stressful life events have been implicated in the onset and course of various illnesses (9). Long-term stress has harmful effects on an individual's mental and physical health (7). The association between stress and fatigue is a well-documented in the general population (10). Fatigue has become an issue of concern among employees resulting from prolonged life stressors (11). Previous research has demonstrated that work-related stressors contribute to an individual's stress level and can lead to unfavorable health consequences (8). Many people with fatigue report having experienced a very stressful situation prior to the manifestation of the disease, with some cases describing a concomitant infection or other determining factors (12). The role of psychosocial factors in the development of fatigue would be better demonstrated by examining the predictive effects of recent stressful events (13).

Nowadays, highly demands and stressful work situations have become common in modern work (14, 15). Working adults may experience diverse type of stressful life events, which are quite different from the general population. Previous studies have shown that family-related life events occur most frequently in the general population (16). In the working adults, except for family-related stressors, work-related stressors are also very common (17). Currently, evidence on the role of stressful life events in fatigue among the Chinese working adults is lacking. There is a lack of studies comparing the impact of different stressors on fatigue, we are not sure if some specific types of life events (such as work-related stressors) were more potent on fatigue than others. Also, there are no direct tests of the stressor-disease specificity hypothesis, we are not sure if specific types of events linked to specific diseases (such as the association between in love or engagement and fatigue) (4, 18, 19) found that negative life events could influence short-term fluctuations, producing an increase in symptoms of fatigue but with recovery to pre-stress levels when the event is resolved or coping mechanisms are activated to reduce the negative impact (9). However, the effects of positive life events on fatigue are not clear enough.

As an Asian country deeply influenced by traditional culture, China is relatively partial to authoritarianism, collectivism, and kinship, which is different from the horizontal organization and rationalism that popular in Western countries (20). The Chinese workers are hardworking and obedient due to the social culture of nationalism, stability, and harmony (21). In western countries, such as the UK and USA, workers share a lower level of collectivism and power distance when compared with Asian countries, the psychosocial work environment they experienced were better than most Chinese workers (21). Although many Chinese companies or organizations have been working hard to improve the psychosocial work environment of workers, the workload of full-time workers has not decreased. Most industries face a major challenge in maintaining a healthy and productive workforce, it is necessary to explore the associations between stressful life events and fatigue among Chines working adults. The current study aimed at exploring the possible prospective associations between the cumulative and specific stressful life events and self-reported fatigue among Chinese government employees.

## Materials and Methods

### Ethics Statement

This study was approved by the Human Research Ethics Committee of Central South University. Written informed consent was obtained before investigation was conducted.

### Participants

Employees were consecutively recruited if they (a) were government employees who worked in Hunan province. (b) were aged between 18 to 60 years old.

### Procedure

The present analysis is based on the Cohort Study on Chronic Diseases of Government Employees in Hunan Province, which was carried out in five cities of Hunan Province, China, between January 2018 and May 2021 (22). A total of 32 public sectors or organizations were recruited in the five selected cities. Once a public sector or organization is agreed to participate in our project, all employees in that public sector or organization was invited to the cooperated general hospital in their located site, complete the digital questionnaire. In this program, a digital self-reported questionnaire platform was established to collect information on employees. Recruited participants accessed the questionnaire with URLs sent by Short Messaging Service (SMS) and answered the questions via cellphone, tablet, or PC. Following written informed consent, employees were enrolled in the study and completed study questionnaires. All the government employees (*n* = 20863) who worked in the 32 public sectors or organizations were recruited, 2838 of them refused to participate in our project, 18025 government employees were enrolled in the project. In the current study, 1819 of them were excluded due to lack of demographic and fatigue-related data, 16206 participants were finally included at baseline, 13,668 of them were followed. See Supplementary Figure S1 for the details.

## Measures

### Stressful Life Events

Life events were assessed by the Life Events Scale (LES) (23), in which a total of 48 items are classified into several dimensions: family/marriage, work/study, health status, economic problems, accidents and legal disputes. Life events involve serious illness, housing, financial crises, relationship breakdowns, job satisfaction, social support in the workplace, etc. Participants can choose “yes” or “no” for each event based on their experience over the past year. The Chinese version of LES was used in this study. This self-report scale was widely used in China, which has reported favorable validity and reliability (24, 25). Based on previous research (16, 26), we divided the number of life events as four groups: none, one, two and three or more life-events. In addition, the participants were asked to report the severity level of each event. The severity score was divided into four groups: 1 = mild impact, 2 = moderate impact, 3 = severe impact, 4 = extreme severe impact. To obtain a cumulative severity score of all events, the sum of the severity scores of each life event was calculated (25). In this study, the cumulative severity score was grouped into four groups: zero points, 1-3 points, 4-7 points and ≥8 points.

Besides, these 48 life events were classified into five groups: events related to family & marriage, events related to work, events related to economic problems, events related to accidents and legal disputes and events related to health. According to the nature of these events, they were also divided into positive life events and negative life events in the current study (27). These different types of life events were grouped into two groups: none and one or more events.

### Chronic Fatigue

We determined fatigue by using a single item, measuring the frequency of its occurrence. Previous studies showed that correlation patterns and multivariate analysis revealed a strong and significant association between the single-item measure and the other scale analysis on fatigue (28–30). In this study, the outcome variable “perceived fatigue status” was evaluated by self-report, with the response options consisting of “not at all,” “sometimes” and “often” on a Likert-type three-point scale.

Participants were asked by the question “With what frequency have you felt fatigue over the past 3 months?” Participants rated the items on a 3-Likert scale with response options of not at all, sometimes and often, with a score ranged from 0–2. A higher score indicates greater perceived fatigue. A cut-off score of ≥2 denotes perceived fatigue in the current study (28, 29, 31).

### Covariates

Some variables included gender, age, marital status (married, single/divorced/widowed), household income, educational achievement (primary school, secondary school/university/university or above), working hours per day, level of employment (low/intermediate/high), type of work (cognitive tasks / manual tasks), self-reported chronic disease (yes/no) and sleep quality. In the current study, sleep quality was assessed by the 19-item Pittsburgh Sleep Quality Index (PSQI). Clinically, Poor sleep quality was defined by a PSQI global score >5 (32). The details of sleep quality measurement were introduced in our previous research (22). Some behavior factors including alcohol consumption (never/occasional drinking, former drinkers, current drinkers), smoking status (never/occasional, former smokers, current smokers), attending business dinner frequently (yes/no) were included too. In this study, alcohol use was defined as drinking alcohol at least once a week for at least half a year. Smoking is defined as smoking at least one cigarette a day for at least half a year. Attending business dinner frequently is defined as attending business dinner more than once a week over the past year.

### Analytic Plan

The mean and standard deviation (SD) or proportion (%) of covariate characteristics were presented among participants with or without self-reported fatigue. Chi-square tests were used for categorical variables. Logistic regression models were used to estimate ORs and corresponding 95% confidence intervals (CIs) of self-reported fatigue for different type of stressful life events. In the logistic regression models, each model included known and potential confounders (multicollinearity diagnosis between those variables were conducted, collinearity between life events and different covariates were not found). Model 1 adjusted for gender and age. Model 2 further adjusted for some baseline factors (including marital status, education level, family income, grades of employment, type of work, work hours per day, sleep quality and whether have chronic disease) and model 3 further included behavioral factors (including smoking status, drinking status and attending business dinner frequently or not). To examine associations of worked-related stressors and self-reported fatigue in different participants, analyses were conducted across subgroups stratified by gender and age. Please see Supplementary data for the details.

Data analyses were conducted by SPSS 26.0. All p-values refer to two-tailed tests. A *p*-value less than 0.05 was considered statistically significant.

## Results

### Descriptive Statistics

Among the 16,206 participants who were finally included at baseline, 13,668 of them were followed. After the exclusion of missing demographic and fatigue-related data, 16,206 government employees at baseline and 12,639 government employees at follow up were available for the final analysis. Of those participants, 11,658 government employees (4,050 males and 7,608 females, with a mean age of 38.09 years) were eligible for the longitudinal analyses after excluding participants who suffered from fatigue at baseline. Please see Supplementary Figure S1 for the details.

The mean age of the sample was 37.11 years (S.D = 9.53), most of them were younger than 40 years old (66.23%) and 61.52% of the sample were female. More than two thirds of the participants (68.78%) had a college education and most of them (78.03%) were currently married or cohabitating with someone. Also, 9.86% of the included government employees were current drinkers and 11.49% of them were current smokers. Of the included Chinese government employees, 7.63% reported that they perceived fatigue (fatigue score ≥2) over the past 3 months, 70.47% reported that they have experienced stressful life events over the past year. Of the Chinese government employees who reported stressful life events, 60.45% reported that they experienced negative stressful life events and 43.87% reported that they experienced positive stressful life events over the past year. See Table 1 and the supplementary data for the details.

**Table 1:** Socio-demographic characteristics, stressful life events between employees with/without chronic fatigue at baseline (*n* = 16206).

**Variables**	**Total (*n*/%)**	**Self-reported fatigue**		**χ^2^**	***p***
		**No**	**Yes**		
**Gender**				30.926	<0.001
Male	6238 (38.48)	5847	391		
Female	9968 (61.52)	9104	864		
**Age (year)**				23.137	<0.001
18-30	4908 (30.29)	4529	379		
31–40	5824 (35.94)	5309	515		
41–50	3551 (21.91)	3300	251		
51–60	1923 (11.86)	1813	110		
**Marital status**				7.674	0.022
Single	3184 (19.65)	2966	218		
Married	12647 (78.03)	11649	998		
Divorced/Widowed	375 (2.32)	336	39		
**Education**				14.405	0.001
High school or below	960 (5.59)	904	56		
University	11147 (68.78)	10226	921		
Graduate	4099 (25.29)	3821	278		
**Family income per year (RMB)**				6.237	0.044
<100000	7361 (45.42)	6749	612		
110000 300000	7875 (48.59)	7300	575		
> 300000	970 (5.99)	902	68		
**Grades of employment**				6.693	0.031
Low	7824 (48.28)	7183	641		
Intermediate	5570 (34.37)	5143	427		
High	2812 (17.35)	2625	187		
**Type of work**				13.906	<0.001
Cognitive tasks	9116 (56.25)	8473	643		
Manual tasks	7090 (43.75)	6478	612		
**Working hours per day (hour)**				62.571	<0.001
<4	274 (1.69)	256	18		
4–6	1058 (6.53)	999	59		
7–8	10198 (62.93)	9502	696		
>8	4676 (28.85)	4194	482		
**Smoking**				0.229	0.632
No	14344 (88.51)	13228	1116		
Yes	1862 (11.49)	1723	139		
**Drinking**				0.182	0.670
No	14609 (90.14)	13482	1127		
Yes	1597 (9.86)	1469	128		
**Attending business dinner frequently**				11.899	0.001
No	14200 (87.62)	13139	1061		
Yes	2006 (12.38)	1812	194		
**Self-reported chronic disease**				89.562	<0.001
No	13829 (85.33)	12872	957		
Yes	2377 (14.67)	2079	298		
**Sleep quality**				879.506	<0.001
Good ( ≤ 5)	10258 (63.29)	9950	308		
Poor (>5)	5948 (36.71)	5001	947		
**Number of life events**				438.707	<0.001
0	5086 (29.53)	4917	169		
1	2784 (16.17)	2654	130		
2	2416 (14.03)	2283	133		
≥3	6935 (40.27)	6053	882		
**Severity score of life events**				633.628	<0.001
0	5091 (29.56)	3088	169		
1–3	4841 (28.11)	2353	226		
4–7	3531 (20.50)	1390	292		
≥8	3758 (21.83)	980	267		

### Comparisons on the Key Variables by the Covariates

As presented in Table 1, being female, younger age, higher education levels, lower family income, divorced or widowed, lower grade of employment, have a physical oriented work, have a longer working hours per day, poor sleep quality, attending business dinner frequently, and had chronic disease were significantly associated with self-reported fatigue (*p* < 0.05). Non-significance was found between smoking status and drinking status (*p* < 0.05).

### Cross-Sectional Associations Between Cumulative Number of Life Events, Cumulative Life Events Severity Score and Self-Reported Fatigue

The results for associations between cumulative number of life events and self-reported fatigue showed that experiencing two life events at baseline was associated with a statistically significant 1.31 (95% CI: 1.05–1.69) times greater likelihood of self-reported fatigue at baseline. Employees with more than three life events had 2.58 (95% CI: 2.14–3.11) times greater likelihood of self-reported fatigue in the full-adjusted model. The associations between experiencing one life events and self-reported fatigue were not statistically significant (OR = 1.14; 95% CI: 0.90–1.46).

The results for associations between cumulative life events severity score and self-reported fatigue showed that 4–7 points and ≥8 points group were positive associated with self-reported fatigue in the past 3 month. Chinese employees with 4–7 points of severity score had 1.85 (95% CI: 1.50–2.28) times greater likelihood of self-reported fatigue. Government employees with ≥8 points of severity score had 3.36 (95% CI: 2.76–4.10) times greater likelihood of self-reported fatigue over the past 3 months. For 1–3 points group, the association was not statistically significant in the fully-adjusted model (OR = 1.16; 95% CI: 0.94–1.42). See Table 2 for the details.

**Table 2:** Cross-Sectional associations between cumulative number of life events, cumulative life events severity score and self-reported fatigue.

	**Model 1** ^**a**^		**Model 2** ^**a**^		**Model 3** ^**a**^	
	**OR**	**95%CI**	**OR**	**95%CI**	**OR**	**95%CI**
**Number of life events**						
0 (reference)						
1	1.43*	(1.12–1.82)	1.14	(0.90–1.45)	1.14	(0.90–1.46)
2	1.69*	(1.33–2.15)	1.32*	(1.02–1.68)	1.31*	(1.05–1.69)
≥3	4.31*	(3.61–5.14)	2.58*	(2.14–3.10)	2.58*	(2.14–3.11)
**Severity score of life events**						
0 (reference)						
1-3	1.45*	(1.17–1.79)	1.16	(0.93–1.42)	1.16	(0.94–1.42)
4-7	2.70*	(2.21–3.31)	1.84*	(1.49–2.29)	1.85*	(1.50–2.28)
≥8	6.08*	(5.05–7.31)	3.37*	(2.77–4.14)	3.36*	(2.76–4.10)

*p < 0.05.

*^a^Model 1 adjusted for age and gender; Model 2 further adjusted for marital status, education level, family income, grades of employment, type of work, work hours per day, sleep quality and whether have chronic disease; Model 3 further adjusted for smoking, drinking and attending business dinner frequently or not*.

### Longitudinal Associations Between Cumulative Number of Life Events, Cumulative Life Events Severity Score and Self-Reported Fatigue

The results for associations between cumulative number of life events and self-reported fatigue showed that experiencing one life events at baseline was associated with a statistically significant 1.42 (95% CI: 1.06–1.92) times greater likelihood of self-reported fatigue at follow up. Also, experiencing two life events at baseline was associated with a statistically significant 1.65 (95% CI: 1.22–2.21) times greater likelihood of self-reported fatigue at follow-up. Employees with more than three life events at baseline had 2.57 (95% CI: 2.02–3.24) times greater likelihood of self-reported fatigue at follow-up in the full-adjusted model.

The results for associations between cumulative life events severity score at baseline and self-reported fatigue at follow-up showed that higher severity score was associated with higher incidence of self-reported fatigue. Chinese employees with 1–3 points of severity score had 1.43 (95% CI: 1.10–1.85) times greater likelihood of self-reported fatigue. Also, employees with 4–7 points of severity score had 2.56 (95% CI: 1.97–3.28) times greater likelihood of self-reported fatigue. Government employees with ≥8 points of severity score had 2.72 (95% CI: 2.08–3.51) times greater likelihood of self-reported fatigue at follow-up. See Table 3 for the details.

**Table 3:** Longitudinal associations between cumulative number of life events, cumulative life events severity score and self-reported fatigue.

	**Model 1** ^**a**^		**Model 2** ^**a**^		**Model 3** ^**a**^	
	**OR**	**95%CI**	**OR**	**95%CI**	**OR**	**95%CI**
**Number of life events**						
0 (reference)						
1	1.52*	(1.14–2.04)	1.44*	(1.07–1.93)	1.42*	(1.06–1.92)
2	1.78*	(1.33–2.40)	1.66*	(1.23–2.23)	1.65*	(1.22–2.21)
≥3	3.05*	(2.43–3.82)	2.61*	(2.06–3.30)	2.57*	(2.02–3.24)
**Severity score of life events**						
0 (reference)						
1-3	1.52*	(1.18–1.96)	1.44*	(1.11–1.86)	1.43*	(1.10–1.85)
4-7	2.90*	(2.26–3.72)	2.59*	(2.01–3.34)	2.56*	(1.97–3.28)
≥8	3.35*	(2.61–4.30)	2.76*	(2.13–3.59)	2.72*	(2.08–3.51)

*p < 0.05.

*^a^Model 1 adjusted for age and gender; Model 2 further adjusted for marital status, education level, family income, grades of employment, type of work, work hours per day, sleep quality and whether have chronic disease; Model 3 further adjusted for smoking, drinking and attending business dinner frequently or not*.

### Longitudinal Associations Between Different Type of Life Events at Baseline and Self-Reported Fatigue at Follow-Up

In the full-adjusted model, experiencing negative life events at baseline was associated with a statistically significant 2.06 (95% CI: 1.69–2.51) times greater likelihood of self-reported fatigue at follow-up. However, experiencing positive life events at baseline were not associated with self-reported fatigue at follow-up (OR: 0.93, 95%CI: 0.78–1.11).

When classified into different dimensions, the results showed that two types of life events including work-related events (OR = 1.87, 95%CI: 1.56–2.25), economic-related events (OR = 1.28, 95%CI: 1.06–1.55) were significantly associated with a higher likelihood of self-reported fatigue. Family/marriage-related events (OR = 0.98, 95%CI: 0.82–1.17), health-related events (OR = 1.21, 95%CI: 0.94–1.57), accident / legal dispute-related events (OR = 1.03, 95%CI: 0.81–1.32) were not associated with self-reported fatigue. See Table 4 and Supplementary Table S2 and for the details.

**Table 4:** Longitudinal associations between different type of life events at baseline and self-reported fatigue at follow-up.

**Category**	**Model 1** ^**a**^		**Model 2** ^**a**^		**Model 3** ^**a**^	
	**OR**	**95%CI**	**OR**	**95%CI**	**OR**	**95%CI**
**Type of life events**						
Number of negative life events (0 *vs*. ≥1)	2.41*	(1.99–2.92)	2.06*	(1.69–2.51)	2.06*	(1.69–2.51)
Number of positive life events (0 *vs*. ≥1)	0.94	(0.77–1.14)	0.93	(0.78–1.11)	0.93	(0.78–1.11)
**Specific life events**						
Number of events related to family & marriage (0 *vs*. ≥1)	1.04	(0.87- 1.24)	0.98	(0.82–1.17)	0.98	(0.82–1.17)
Number of events related to work (0 *vs*. ≥1)	1.96*	(1.64–2.33)	1.87*	(1.56–2.25)	1.87*	(1.56–2.25)
Number of events related to economic problems (0 *vs*. ≥1)	1.34*	(1.11- 1.62)	1.28*	(1.06–1.55)	1.28*	(1.06–1.55)
Number of events related to accidents and legal disputes (0 *vs*. ≥1)	1.13	(0.89–1.44)	1.03	(0.81–1.32)	1.03	(0.81–1.32)
Number of events related to health (0 *vs*. ≥1)	1.40*	(1.08–1.79)	1.22	(0.94–1.57)	1.21	(0.94–1.57)

*p < 0.05.

*^a^Model 1 adjusted for age and gender; Model 2 further adjusted for marital status, education level, family income, grades of employment, type of work, work hours per day, sleep quality and whether have chronic disease; Model 3 further adjusted for smoking, drinking and attending business dinner frequently or not*.

### Longitudinal Associations Between Specific Life Events at Baseline and Self-Reported Fatigue at Follow-Up

Among the 48 major life events, work stress, not satisfied with the current job, self-pregnancy or wife pregnancy, in love or engagement, new member was added to the family and other events shown in Table 5 are the top 10 life events in terms of prevalence of events among our participates (see Supplementary Table S1). The relationship between these most common life events at baseline and self-reported fatigue at follow-up were tested in different models. Regarding the specific stressful life events, work stress (OR = 1.76, 95%CI: 1.45–2.13), as well as not satisfied with the current job (OR = 1.95, 95%CI: 1.58–2.40), in debt (OR: 1.75, 95%CI: 1.40–2.17) were significantly associated with a higher likelihood of self-reported fatigue. Self-pregnancy or wife pregnancy (OR = 1.14, 95%CI: 0.86–1.50), in love or engagement (OR = 1.02, 95%CI: 0.78–1.32), new member was added to the family (OR: 1.04, 95%CI: 0.79–1.38), discord with spouse's parents (OR: 0.91, 95%CI: 0.68–1.20), separation from spouse due to work demands (OR: 1.04, 95%CI: 0.80–1.34) and bad relationship between spouse (OR: 0.95, 95%CI: 0.71–1.27) were non-significant factors. The economic situation has improved significantly (OR: 0.62, 95%CI: 0.46–0.85) at baseline was significantly associated with lower incidence of self-reported fatigue at follow-up. See Table 5 for the details.

**Table 5:** Longitudinal associations between specific life events at baseline and self-reported fatigue at follow-up.

**Category**	**Model 1** ^**a**^		**Model 2** ^**a**^		**Model 3** ^**a**^	
	**OR**	**95%CI**	**OR**	**95%CI**	**OR**	**95%CI**
**Specific life events**						
Work stress	1.90*	(1.58–2.29)	1.76*	(1.45–2.13)	1.76*	(1.45–2.13)
Not satisfied with the current job	2.03*	(1.65–2.50)	1.95*	(1.58–2.40)	1.95*	(1.58–2.40)
Self-pregnancy or wife pregnancy	1.16	(0.89–1.52)	1.14	(0.86–1.49)	1.14	(0.86–1.50)
In love or engagement	1.01	(0.78–1.30)	1.03	(0.79–1.33)	1.02	(0.78–1.32)
New member was added to the family	1.06	(0.80–1.40)	1.04	(0.79–1.38)	1.04	(0.79–1.38)
In debt	1.84*	(1.48–2.29)	1.75*	(1.41–2.19)	1.75*	(1.40–2.17)
Discord with spouse's parents	0.95	(0.71–1.24)	0.91	(0.69–1.21)	0.91	(0.68–1.20)
Separation from spouse due to work demands	1.06	(0.82–1.36)	1.04	(0.80–1.35)	1.04	(0.80–1.34)
Bad relationship between spouse	1.04	(0.77–1.38)	0.96	(0.72–1.28)	0.95	(0.71–1.27)
The economic situation has improved significantly	0.63*	(0.64–0.86)	0.63*	(0.46–0.86)	0.62*	(0.46–0.85)

*p < 0.05.

*^a^Model 1 adjusted for age and gender; Model 2 further adjusted for marital status, education level, family income, grades of employment, type of work, work hours per day, sleep quality and whether have chronic disease; Model 3 further adjusted for smoking, drinking and attending business dinner frequently or not*.

### Adjusted Odds Ratios of Work-Related Life Events at Baseline for Self-Reported Fatigue Stratified by Gender and Age in the Fully Adjusted Model

In Supplementary Table S3, we examined the association between all the work-related life events and self-reported fatigue. The adjusted ORs of self-reported fatigue at follow-up for work-related life events at baseline stratified by gender and age in the fully adjusted model. Regarding the specific work-related stressful life events, work stress was significantly associated with a higher likelihood of self-reported fatigue in both male (OR = 2.00, 95%CI: 1.51–2.64) and female (OR = 1.87, 95%CI: 1.55–2.24). In addition, not satisfied with the current job was significantly associated with a higher likelihood of self-reported fatigue in both male (OR = 1.48, 95%CI: 1.14–1.91) and female (OR = 1.76, 95%CI: 1.49–2.08). Discord with colleagues was significantly associated with a higher likelihood of self-reported fatigue in both male (OR = 1.48, 95%CI: 1.14–1.91) and female (OR = 1.76, 95%CI: 1.49–2.08). Additionally, male employees who reported discord with colleagues reported a higher likelihood of self-reported fatigue (OR = 1.72 and 2.14, respectively). For different age group, work stress was significantly associated with a higher likelihood of self-reported fatigue in age group 18–30, age group 31–40 and age group 41–50 (OR = 1.91, 2.07 and 1.90, respectively). Not satisfied with the current job was significantly associated with a higher likelihood of self-reported fatigue in age group 18–30, age group 31–40 and age group 41–50 (OR = 1.67, 1.62, 1.92 and 2.30, respectively) younger employees (younger than 40) who reported discord with colleagues reported a higher likelihood of self-reported fatigue (OR = 1.72 and 2.14, respectively).

## Discussion

### Key Findings

Of the included 16,206 Chinese government employees at baseline, 60.68% reported that they experienced negative stressful life events and 44.02% reported that they experienced positive stressful life events. The results of this study showed that cumulative stressful life events experienced at baseline were positively associated with self-reported fatigue at follow-up. After adjusting sociodemographic factors, occupational factors and health behavior related factors, negative life events were significantly associated with self-reported fatigue. Some specific life events including events related to work and events related to economic problems were significantly associated with self-reported fatigue. Specifically, work stress (OR = 1.76, 95%CI: 1.45–2.13), as well as not satisfied with the current job (OR = 1.95, 95%CI: 1.58–2.40), in debt (OR = 1.75, 95%CI: 1.40–2.17) were significantly associated with self-reported fatigue. The economic situation has improved significantly (OR=0.62, 95%CI: 0.46–0.85) at baseline is a protective factor for self-reported fatigue, which was significantly associated with lower incidence of self-reported fatigue at follow-up.

### Comparison With Previous Research

Previous studies have reported on the adverse effects of stressful life events on fatigue (9, 13, 33, 34). In the current study, we found that Chinese government employees who experienced more than 3 life events over the past year was most strongly related to high fatigue levels. Also, higher severity score of stressful life events was associated with higher level of fatigue among Chinese government employees. Which was consistent with previous studies (35). We sought to determine, in a population-based sample of Chinese working adults, whether subjective fatigue differed according to the type of life event that precipitated them. The results showed that negative stressful life events were associated with greater likelihood of subjective fatigue while the association between positive stressful life events and subjective was not significant among Chinese government employees. Most studies only focused on the effect of negative life events (9, 33, 36) on fatigue and did not consider the difference between different type of life events. This study is the first attempt to show that positive life events are not associated with fatigue among Chinese working adults. Compared with negative life events, positive stressful life events were more predictable and acceptable, which may be easier to adapt to and manage with (19). The prolonged activation model suggests that next to stressors itself, cognitive anticipation of a stressor can lead to stress-related physiological activity, insufficient recovery, and ill health (37). It is said that there were three forms of anticipation, including positive anticipation, negative anticipation and positive outcome expectancy (10). Employees being preoccupied with work in a negative way (i.e., negative anticipation of a stressful work situation) has a negative impact on fatigue (38). For those companies or organizations, it is important to identify working adults who have experienced negative life stressors and provide effective interventions (such as exercise, cognitive behavioral therapy) (31).

Previous research demonstrated that the effects of life events consistently differed, depending on the category of stressful life events (39). The analysis of the most common separate items in this study suggested that work-related life events including perceived work stress, not satisfied with the current job and economic-related life events including in debt that were the key components. Experienced work-related stressors may mean the psychosocial work environment of those employees were poor, which would contribute to high work demands and low social support, resulted in fatigue (8, 11, 14, 15, 38, 40). In Gimeno et al. study (12), people experienced some economic-related life events usually reported higher level of fatigue. Experiencing economic-related life events may influence the ability of employees to balance work and personal life (41). When people experienced some economic-related or family-related life events, the ability and willingness to lower the boundaries of personal life are lower, and the degree of penetration of work into personal life is higher, personal life may easily interfere with work and resulted in fatigue (41). Previous studies showed that people who experienced health problems were easier to perceived fatigue (6). The associations between health- related events and fatigue was not statistically significant in this study, which need further exploration.

There are fewer studies addressing the extent to which gender differences in exposure to stressful life events are associated with differential vulnerability to illnesses (19). It is said that female is generally expected to carry the load of both work and family, working women work a second shift which starts when they reach home (42), which increases their burden and stress level (34). Additionally, it is believed that females were more sensitive to what happens in their social networks, so they exposed to more interpersonal stressful life events than male (19, 43). However, the results in the current study showed that no significant difference between gender were found. It may be that with the changes in society in the past few decades, the gender inequality in housework and work among Chinese workers has decreased. We think further research is needed in the future. In addition, we found that younger employees who reported work-related life events were significantly associated with higher level of fatigue than older employees. The possible reason is that young employees generally have shorter working years and a lower level of occupation. They usually have a heavier workload and are more likely to work long hours than older employees. Therefore, they are more likely to be exposed to work-related life events and subjective fatigue (44–46).

### Future Implications

The current study demonstrated that work-related life events and economic-related life events were highly associated with fatigue among Chinese government employees. Given that many government employees have experienced multiple life events and the negative effects of those life events were statistically significant, it would be important to explore the work and personal life boundary management in Chinese working adults (41). Understanding the boundaries surrounding the work and personal life domains may contributed to a better health outcome on working adults, and improve their work efficiency (47).

Although the situation is changing in recent years, some countries (such as China and South Korea) in Asia are relatively partial to authoritarianism, collectivism, and kinship, which is different from the horizontal organization and rationalism that popular in Western countries (20). The Chinese workers are hardworking and obedient due to the social culture of nationalism, stability, and harmony. Thus, we believe the negative impact of work-related stressors among Chinese workers especially for those working in the government was particularly significant. We think it is necessary to study the influence of social culture on stress management and health among the working population in developing countries, which is helpful to develop effective interventions with better cultural sensitivity.

Currently, Cohen et al. indicated in their study that some important questions for which we lack adequate evidence are whether previous stressors or ongoing chronic stressors moderate responses to current ones and whether the stress load accumulates with each additional new stressor (19). Understanding the nature of the cumulative effects of stressors may be key to obtaining sensitive assessments of the effects of stressful life events on different disease and for planning effective interventions to reduce the impact of stressful life events on individuals' health (19). Lastly, we believe that possible underlying physiological and psychosocial causal pathways leading to fatigue have rarely been advanced and need to be examined more comprehensively (45).

### Limitations

The present study has some limitations. First, we have classified those stressful life events into different types according to previous research (27), but participants may feel different about these stressors. In the future, it is necessary to further ask these participants to evaluate the event they have experienced is a positive event or a negative event, which may help the researchers better explore the relationship between stressful life events and fatigue. Second, it is possible that the one-item measure of fatigue in the current study could not capture the multidimensional nature of the fatigue experience (48). Thus, we cannot completely exclude misclassification bias as a source of error that explains the current observed associations. Finally, although we adjusted for a number of potential confounders, the results in this study might still be affected by unmeasured or residual confounding (especially genetic background as well as social support and coping style) (49).

## Conclusion

This study showed that cumulative number of stressful life events experienced at baseline were positively associated with self-reported fatigue at follow-up. After adjusting some background factors and health behavior related factors, negative life events were significantly associated with self-reported fatigue. Some specific life events including work stress, job dissatisfaction and in debt were associated with self-reported fatigue. When stratified by gender and age, younger employees who experienced work-related stressors were more vulnerable to self-reported fatigue. Effective interventions should be provided to working adults who have experienced stressful life events, especially negative life events.

## Data Availability Statement

The raw data supporting the conclusions of this article will be made available by the authors, without undue reservation.

## Ethics Statement

The studies involving human participants were reviewed and approved by the Human Research Ethics Committee of Central South University. The patients/participants provided their written informed consent to participate in this study.

## Author Contributions

Study design: DQ and SX. Data collection: DQ, YL, RL, JH, DL, and FO. Data analysis and interpretation: DQ, YL, and SX. All authors were involved in writing the paper and had final approval of the submitted and published versions.

## Funding

This research was supported by the Ministry of Science and Technology of China (Grant No: 2016YFC0900802). The funding agency did not take part in the design of the study and collection, analysis, and interpretation of data and in writing the manuscript.

## Conflict of Interest

The authors declare that the research was conducted in the absence of any commercial or financial relationships that could be construed as a potential conflict of interest.

## Publisher's Note

All claims expressed in this article are solely those of the authors and do not necessarily represent those of their affiliated organizations, or those of the publisher, the editors and the reviewers. Any product that may be evaluated in this article, or claim that may be made by its manufacturer, is not guaranteed or endorsed by the publisher.

## Abbreviations

SMS: Short Messaging Service
PSQI: The Pittsburgh Sleep Quality Index
LES: The Life Events Scale
CI: confidence interval
OR: odds ratio.
