# Supplementary material for: Stressful Life Events and Chronic Fatigue Among Chinese Government Employees: A Population-Based Cohort Study
Source: Front Public Health. 2022 Jul 7;10:890604. doi: 10.3389/fpubh.2022.890604 (PMC9300904; doi:10.3389/fpubh.2022.890604)
Supplement: Supplementary file 1 [file Data_Sheet_1.PDF]

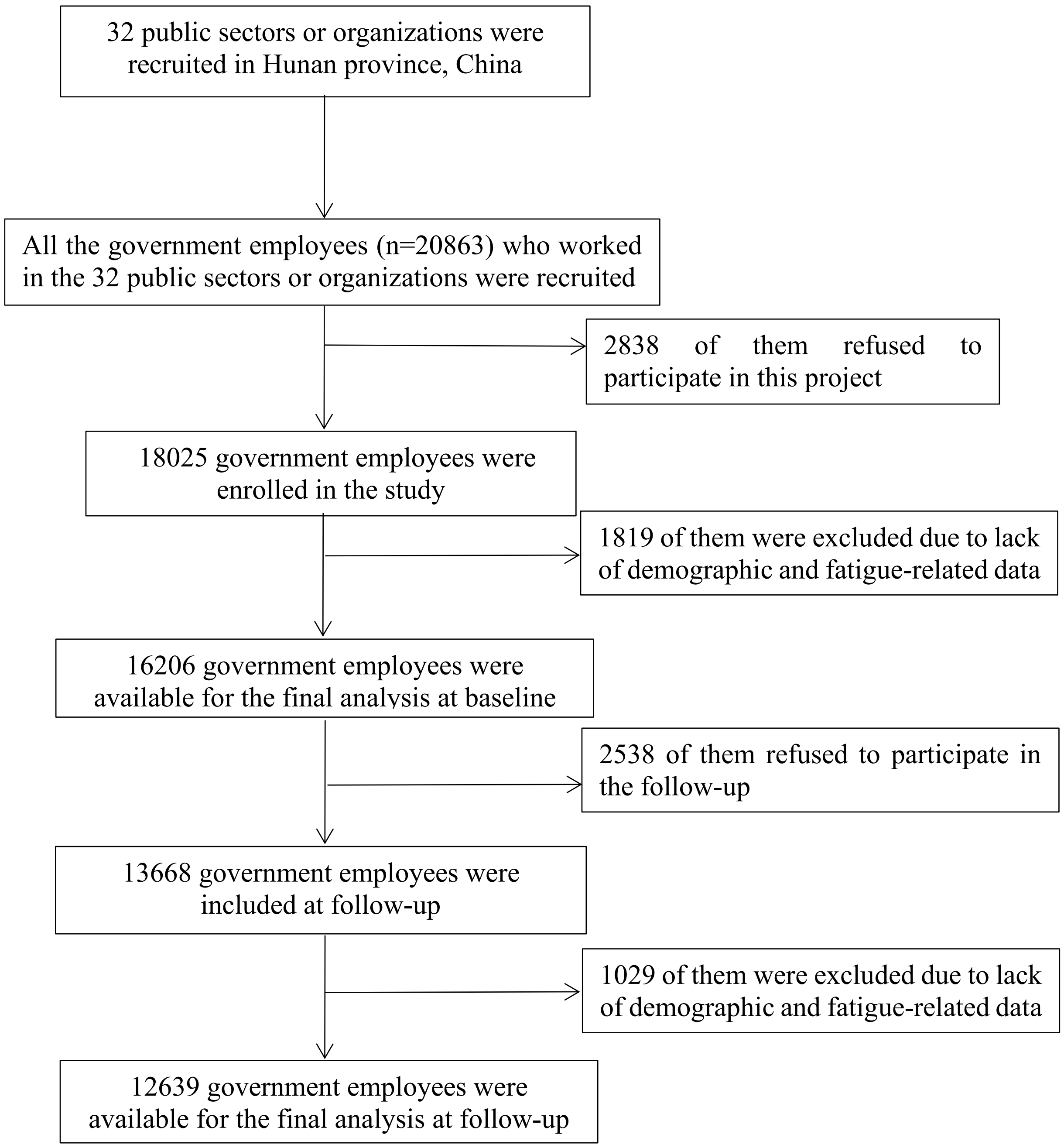
32 public sectors or organizations were recruited in Hunan province, China
All the government employees (n=20863) who worked in the 32 public sectors or organizations were recruited
2838 of them refused to participate in this project
18025 government employees were enrolled in the study
1819 of them were excluded due to lack of demographic and fatigue-related data
16206 government employees were available for the final analysis at baseline
2538 of them refused to participate in the follow-up
13668 government employees were included at follow-up
1029 of them were excluded due to lack of demographic and fatigue-related data
12639 government employees were available for the final analysis at follow-up

Fig. S1 Inclusion process for the participants

Table S1 Baseline characteristics of positive and negative life events

Nature of the life events	Item	N	%	Rank of prevalence
**Positive life events**		**7110**	**43.9**	
	Pregnancy or wife pregnancy	2226	13.7	3
	In love or engagement	2178	13.4	4
	Add new members to the family	2067	12.8	5
	The economic situation has improved significantly	1420	8.8	10
	Promotion	1236	7.6	14
	Get married	998	6.2	18
	Marital relationship becomes better	975	6.0	19
	start working	783	4.8	23
	Outstanding personal achievement	667	4.1	26
**Negative life events**		**9797**	**60.5**	
	Work stress	4469	27.6	1
	Not satisfied with the current job	2370	14.6	2
	In debt	1856	11.5	6
	Discord with spouse's parents	1844	11.4	7
	Separation from spouse due to work demands	1704	10.5	8
	Bad relationship between spouse	1567	9.7	9
	Unsatisfied sex life	1345	8.3	11
	Difficulties in child discipline	1313	8.1	12
	Misunderstood, blamed, false accusation, argument	1269	7.8	13
	Family financial difficulties	1231	7.6	15
	Life style changes	1207	7.4	16

	Deduction of bonuses and fines	1066	6.6	17
	Family member seriously ill/ injured	957	5.9	20
	poor relationship with superiors	875	5.4	21
	Broken love	831	5.1	22
	Miscarriage by yourself or wife	689	4.3	24
	Death of family member	672	4.1	25
	Parental discord	644	4.0	27
	Housing shortage	587	3.6	28
	Husband and wife separated due to discord	508	3.1	29
	Children leave home for a long time	418	2.6	30
	Discord with colleagues	363	2.2	31
	Go abroad for the first time	361	2.2	32
	Theft, property damage	328	2.0	33
	Friend died	280	1.7	34
	Friends seriously ill/ injured	267	1.6	35
	divorce	266	1.6	36
	Unexpected frights, accidents, natural disasters	233	1.4	38
	Intervene in civil legal disputes	238	1.5	37
	Failure of children's advancement or employment	214	1.3	39
	Self seriously ill/injured	209	1.3	40
	Unemployed	186	1.1	41
	spouse has an affair	156	1.0	42
	yourself or your spouse undergo sterilization	142	0.9	43
	retired or did not arrange a specific job	132	0.8	44
	examination failed	68	0.4	45
	Detained, tried	55	0.3	46
	Death of spouse	55	0.3	47

	Violation of one child policy	48	0.3	48

Table S2 Baseline characteristics of different types of life events

Type of life events	Item	N	%
**Events related to family & marriage**		**9070**	**56.0**
	In love or engagement	2178	13.4
	Broken love	831	5.1
	Get married	998	6.2
	Pregnancy or wife pregnancy	2226	13.7
	Miscarriage by yourself or wife	689	4.3
	Add new members to the family	2067	12.8
	Discord with spouse's parents	1844	11.4
	Bad relationship between spouse	1567	9.7
	Husband and wife separated due to discord	508	3.1
	Unsatisfied sex life	1345	8.3
	Separation from spouse due to work demands	1704	10.5
	Spouse has an affair	156	1.0
	Marital relationship becomes better	975	6.0
	Violation of one child policy	48	0.3
	Yourself or your spouse undergo sterilization	142	0.9
	Death of spouse	55	0.3
	Divorce	266	1.6
	Failure of children's advancement or employment	214	1.3
	Difficulties in child discipline	1313	8.1
	Children leave home for a long time	418	2.6
	Parental discord	644	4.0
	Family member seriously ill/ injured	957	5.9

	Death of family member	672	4.1
	Go abroad for the first time	361	2.2
	Friends seriously ill/ injured	267	1.6
	Friend died	280	1.7
**Events related to work**		**6628**	**40.9**
	Unemployed	186	1.1
	Start working	783	4.8
	Deduction of bonuses and fines	1066	6.6
	Outstanding personal achievement	667	4.1
	Promotion	1236	7.6
	Job dissatisfaction	2370	14.6
	Work stress	4469	27.6
	Poor relationship with superiors	875	5.4
	Discord with colleagues	363	2.2
	Retired or did not arrange a specific job	132	0.8
	Examination failed	68	0.4
**Events related to economic problems**		**3509**	**21.7**
	Family financial difficulties	1231	7.6
	In debt	1856	11.5
	The economic situation has improved significantly	1420	8.8
	Housing shortage	587	3.6
**Events related to accidents and legal disputes**		**1726**	**10.7**
	Misunderstood, blamed, false accusation, argument	1269	7.8
	Theft, property damage	328	2.0
	Unexpected frights, accidents, natural disasters	233	1.4
	Intervene in civil legal disputes	238	1.5
	Detained, tried	55	0.3
**Events related to health**		**1358**	**8.4**

	Lifestyle changes	1207	7.4
	Self seriously ill/injured	209	1.3

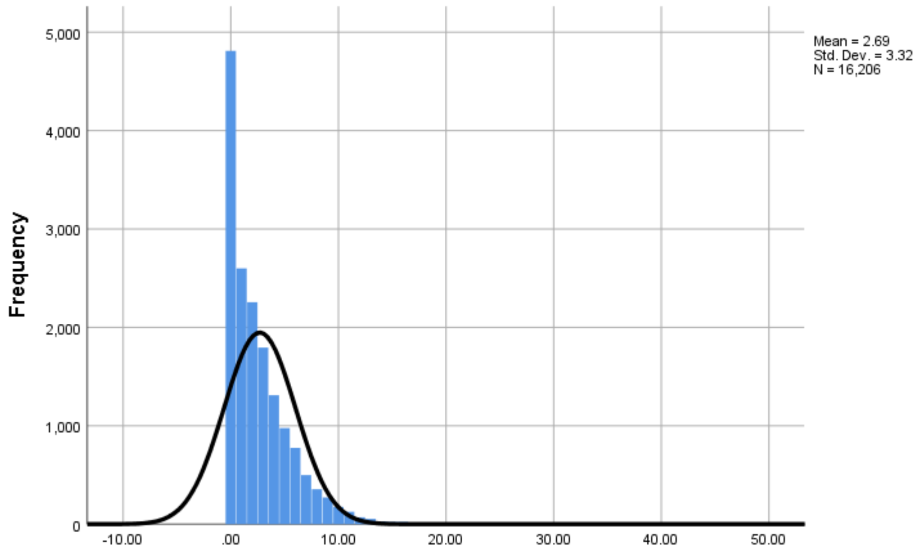

Fig S2 Frequency distribution of number of life events at baseline

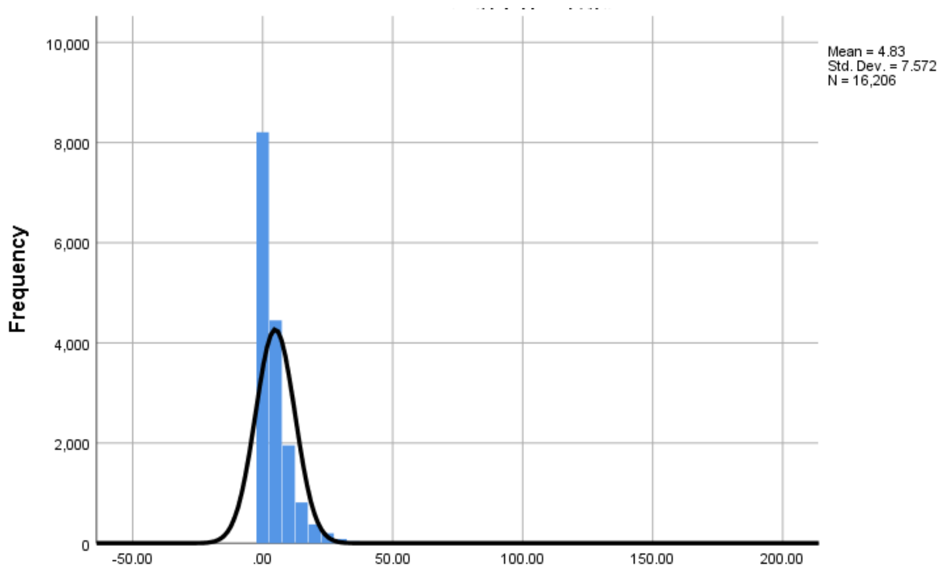

Fig S3 Frequency distribution of score of life events at baseline

Table S3 **Adjusted odds ratios of self-reported fatigue for life events stratified by gender and age in the fully adjusted model** [a]

Work-related life events	Gender		Age			
	Female	Male	18-30	31-40	41-50	≥51
Unemployed	1.07(0.80 - 2.54)	0.74(0.25 - 2.13)	0.93(0.73 - 1.99)	1.19(0.35 - 2.57)	0.96(0.25 - 3.61)	0.74(0.37 - 6.47)
Start working	1.19(0.56 - 1.18)	0.52(0.27 - 1.01)	1.23(0.77 - 1.93)	0.87(0.31 - 2.44)	0.44(0.10 - 1.96)	0.54(0.33 - 3.20)
Failed in exam	0.61(0.22 - 1.99)	1.69(0.46 - 6.23)	0.88(0.40 - 1.94)	0.20(0.03 - 1.03)	1.80(0.94 - 9.37)	0.62(0.09 - 2.23)
Deduction of bonuses and fines	0.97(0.76 - 1.24)	1.93*(1.17 - 3.16)	1.82*(1.22 - 2.70)	1.87*(1.25 - 2.78)	1.79(0.96 - 3.34)	2.99(1.00 - 8.17)
Outstanding personal achievement	0.80(0.72 - 1.58)	1.00(0.50 - 1.96)	1.67(0.82 - 3.36)	0.65(0.80 - 1.33)	0.51(0.14 - 1.91)	1.57(0.56 - 4.37)
Promotion	0.90(0.74 - 1.26)	1.03(0.57 - 1.86)	1.09(0.62 - 1.91)	0.75(0.47 - 1.20)	1.48(0.72 - 3.06)	1.25(0.48 - 3.28)
Job dissatisfaction	1.65*(1.49 - 2.08)	1.80*(1.14 -1.91)	1.64*(1.14 - 2.36)	1.50*(1.05 - 2.12)	2.47*(1.45 - 4.20)	1.99(0.69 - 5.76)
Work stress	1.66*(1.25 - 2.24)	1.48*(1.01 - 2.21)	1.48*(1.05 - 2.09)	1.62*(1.19 - 2.19)	1.80*(1.12 - 2.88)	1.81(0.78 - 4.21)
Bad relationship with superiors	0.97(0.75 - 1.26)	0.89(0.46 - 1.73)	0.82(0.46 - 1.46)	0.78(0.64 - 1.22)	0.83(0.49 - 1.40)	1.70(0.66 - 4.37)
Discord with colleagues	3.80*(2.56 - 5.23)	3.34*(1.62 - 6.92)	1.94*(1.01 - 3.97)	4.98*(2.95 - 8.35)	3.10(1.45 - 6.61)	2.98(0.79 - 7.63)
Retired	0.83(0.43 - 1.97)	1.54(0.55 - 4.26)	1.05(0.58 - 1.89)	2.08(0.99 - 4.62)	1.30(0.29 - 5.70)	0.48(0.13 - 1.82)

Without any life events provided as the reference group; *$p < 0.05$

Abbreviations: CI, confidence interval; OR, odds ratio.

[a] The fully adjusted model adjusted for age, gender, marital status, education level, family income, grades of employment, type of work, work hours per day, sleep quality, whether have chronic disease, smoking, drinking and attending business dinner frequently or not.

Analytic plan

Data analyses were conducted by SPSS 26.0. All p-values refer to two-tailed tests. A p-value less than 0.05 was considered statistically significant.

The mean and standard deviation (SD) or proportion (%) of covariate characteristics were presented among participants with or without self-reported fatigue. Chi-square tests were used for categorical variables.

Logistic regression models were used to estimate ORs and corresponding 95% confidence intervals (CIs) of self-reported fatigue for different type of stressful life events. In the logistic regression models, each model included known and potential confounders. Model 1 adjusted for gender and age. Model 2 further adjusted for baseline social demographic factors (including marital status, education level, family income, grades of employment, type of work, work hours per day, sleep quality and whether have illness) and model 3 further included behavioral factors (including smoking, drinking and attending business dinner frequently or not). Specifically, we conducted seven different analyses (a-g), see Table S5 for the details.

To examine associations of worked-related stress and self-reported fatigue in different participants, analyses were explored across subgroups stratified by gender and age. In addition to covariates, 11 work-related variables were put into the model at the same time in different models.

Table S4 Details of variables controlled by different models

Data Analysis	Model 1	Model 2	Model 3
**a) The associations between cumulative number of life events and self-reported fatigue**	**Outcome: fatigue at baseline**	**Outcome: fatigue at baseline**	**Outcome: fatigue at baseline**
	**Independent variable:** cumulative number of life events,<br>**Covariates:** gender, age	**Independent variable:** cumulative number of life events,<br>**Covariates:** gender, age, marital status, education level, family income, grades of employment, type of work, work hours per day, sleep quality and whether have illness	**Independent variable:** cumulative number of life events,<br>**Covariates:** gender, age, marital status, education level, family income, grades of employment, type of work, work hours per day, sleep quality, whether have illness, smoking, drinking and attending business dinner frequently or not.
**b) the associations between cumulative severity score and self-reported fatigue**	**Outcome: fatigue at baseline**	**Outcome: fatigue at baseline**	**Outcome: fatigue at baseline**
	**Independent variable:** cumulative severity score of life events,<br>**Covariates:** gender, age.	**Independent variable:** cumulative severity score of life events,<br>**Covariates:** gender, age, marital status, education level, family income, grades of employment, type of work, work hours per day, sleep quality and whether have illness	**Independent variable:** cumulative severity score of life events,<br>**Covariates:** gender, age, marital status, education level, family income, grades of employment, type of work, work hours per day, sleep quality, whether have illness, smoking, drinking and attending business dinner frequently or not.
**c) The associations between**	**Outcome: fatigue at follow-up**	**Outcome: fatigue at follow-up**	**Outcome: fatigue at follow-up**

**cumulative number of life events and self-reported fatigue**	**Independent variable:** cumulative number of life events,<br>**Covariates:** gender, age	**Independent variable:** cumulative number of life events,<br>**Covariates:** gender, age, marital status, education level, family income, grades of employment, type of work, work hours per day, sleep quality and whether have illness	**Independent variable:** cumulative number of life events,<br>**Covariates:** gender, age, marital status, education level, family income, grades of employment, type of work, work hours per day, sleep quality, whether have illness, smoking, drinking and attending business dinner frequently or not.
**d) the associations between cumulative severity score and self-reported fatigue**	**Outcome: fatigue at follow-up**	**Outcome: fatigue at follow-up**	**Outcome: fatigue at follow-up**
	**Independent variable:** cumulative severity score of life events,<br>**Covariates:** gender, age.	**Independent variable:** cumulative severity score of life events,<br>**Covariates:** gender, age, marital status, education level, family income, grades of employment, type of work, work hours per day, sleep quality and whether have illness	**Independent variable:** cumulative severity score of life events,<br>**Covariates:** gender, age, marital status, education level, family income, grades of employment, type of work, work hours per day, sleep quality, whether have illness, smoking, drinking and attending business dinner frequently or not.
**e) the associations between different type of life events and self-reported fatigue**	**Outcome: fatigue at follow-up**	**Outcome: fatigue at follow-up**	**Outcome: fatigue at follow-up**
	**Independent variable:** negative life events; positive life events,<br>**Covariates:** gender, age.	**Independent variable:** negative life events; positive life events,<br>**Covariates:** gender, age, marital status, education level, family income, grades of employment, type of work, work hours per day, sleep quality and whether have illness	**Independent variable:** negative life events; positive life events,<br>**Covariates:** gender, age, marital status, education level, family income, grades of employment, type of work, work hours per day, sleep quality, whether have illness, smoking, drinking and attending business dinner frequently or not.
**f) the associations between specific life events and self-reported fatigue**	**Outcome: fatigue at follow-up**	**Outcome: fatigue at follow-up**	**Outcome: fatigue at follow-up**
	**Independent variable:**<br>Events related to family & marriage<br>Events related to work,<br>Events related to economic problems,<br>Events related to accidents and legal disputes,<br>Events related to health,<br>**Covariates:** gender, age.	**Independent variable:**<br>Events related to family & marriage<br>Events related to work,<br>Events related to economic problems,<br>Events related to accidents and legal disputes,<br>Events related to health,<br>**Covariates:** gender, age, marital status, education level, family income, grades of employment, type of work, work hours per day, sleep quality and whether have illness	**Independent variable:**<br>Events related to family & marriage<br>Events related to work,<br>Events related to economic problems,<br>Events related to accidents and legal disputes,<br>Events related to health,<br>**Covariates:** gender, age, marital status, education level, family income, grades of employment, type of work, work hours per day, sleep quality, whether have illness, smoking, drinking and attending business dinner frequently or not.

**g) the associations between specific life events (top 10 life events) and self-reported fatigue**	**Outcome: fatigue at follow-up**	**Outcome: fatigue at follow-up**	**Outcome: fatigue at follow-up**
	**Independent variable:** 48 life events **Covariates:** gender, age.	**Independent variable:** 48 life events **Covariates:** gender, age, marital status, education level, family income, grades of employment, type of work, work hours per day, sleep quality and whether have illness	**Independent variable:** 48 life events **Covariates:** gender, age, marital status, education level, family income, grades of employment, type of work, work hours per day, sleep quality, whether have illness, smoking, drinking and attending business dinner frequently or not.
